# *Equisetum giganteum* influences the ability of *Candida albicans* in forming biofilms over the denture acrylic resin surface

**DOI:** 10.1080/13880209.2017.1321024

**Published:** 2017-04-28

**Authors:** Rafaela Alves da Silva, Laura Pozato Bernardo, Jessica Monique Lopes Moreno, Vanessa Soares Lara, Vinicius Carvalho Porto

**Affiliations:** a Department of Surgery, Stomatology, Pathology and Radiology, Bauru School of Dentistry, University of São Paulo (USP), Bauru, SP, Brazil;; b Bauru School of Dentistry, University of São Paulo (USP), Bauru, SP, Brazil;; c Department of Prosthodontics, Bauru School of Dentistry, University of São Paulo (USP), Bauru, SP, Brazil

**Keywords:** Denture stomatitis, antifungal activity, colony forming units assay, crystal violet, natural products

## Abstract

**Context:***Equisetum giganteum* L. (Equisetaceae) is an endemic plant of Central and South America used in traditional medicine. Natural drugs have been frequently used in the treatment of a myriad of diseases, proving to be an alternative to synthetic chemicals, and have been intensively studied in the prevention of sicknesses, including oral diseases.

**Objective:** This study evaluated the *in vitro* antiadherent activity of *E. giganteum* extract against *Candida albicans* biofilms.

**Materials and methods:** Crystal violet and colony-forming units assays were used to quantify the total biofilm biomass and biofilm living cells on a denture base acrylic resin pretreated with hydroethanolic extract of *E. giganteum* in different concentrations (50, 25, 16, 8, and 4 mg/mL), after 24 h of biofilm development.

**Results:***Equisetum giganteum* affected biofilms by reduction of biomass and living cells per area of acrylic specimens. The results revealed reduction of 15–44% of the biofilm mass and reduction of numbers of colony-forming units (CFUs) present in biofilms (79%) compared to the untreated control (CTRL/PBS). At all concentrations, it demonstrated important antiadherent activity on *Candida albicans* biofilms, the main microbe in denture stomatitis.

**Discussion and conclusion:** The present findings show that *E. giganteum* antimicrobial effects may qualify the extract as a promising natural alternative for topical treatment or prevention of denture stomatitis. The usage of drugs made of natural products shows advantages in relation to synthetic drugs on the market, such as lower cost, lower toxicity, and in relation to the occurrence of microbial resistance.

## Introduction

*Candida albicans* is a microorganism that colonizes the mucosa in a commensal way, yet it may become pathogenic as an opportunist microorganism, being responsible for serious infections in immunosuppressed patients (Odds 1988). The *albicans* is the most abundant and important species from genus *Candida,* currently known as being responsible for human infections, such as vulvovaginitis, nosocomial infections and oral candidiasis (Harwood & Rao 2014).

Several factors contribute to *Candida* pathogenicity, including adhesion to medical devices or host cells, biofilm formation and secretion of hydrolytic enzymes (proteases, phospholipases and hemolysins) (Silva et al. 2011). Successful colonization requires both nonspecific and specific adhesion mechanisms. In the first, the adhesion greatly depends on the apolar component of the microorganism cell wall, while in the latter there is participation of more specific receptors (Harun & Razak 2013). Some *Candida albicans* molecules that mediate cells binding to other cells (host or microbial), inert polymers, or proteins are called adhesins (Cannon & Chaffin 2001). Adhesion and biofilm formation to the denture-fitting surface are critical in the development of denture stomatitis (DS), a superficial form of oral candidiasis that affects 65% of complete denture wearers (Chandra et al. 2001).

The DS treatment requires oral and denture hygiene, including strict disinfection of dentures (Neppelenbroek et al. 2008), removal of dentures at night, correction of denture defects, and utilization of topical and systemic antifungals, such as amphotericin B, nystatin, and azoles (Amanlou et al. 2006; Kulak et al. 1994; Neppelenbroek et al. 2008). Amphotericin B and nystatin are topically used and sometimes applied to the denture-fitting surface before its utilization, while azoles like ﬂuconazole, itraconazole, and ketaconazole are available for systemic antifungal treatment (Ellepola & Samaranayake 1998). Inefficiency of topical antifungal drugs is common, due to dilution and rapid elimination by the saliva ﬂushing action, which may reduce antifungal agents to subtherapeutic concentrations. Furthermore, several doses of these drugs are required, which may reduce the denture adherence to oral tissues. Continued use of systemic antifungal drugs can lead to some limitations regarding drug interactions and toxicity. Moreover, resistant fungal species have evolved due to the spread usage of systemic medications (Amanlou et al. 2006; Pfaller 2012).

Considering the exponentially increasing interest in antimicrobial agents derived from medicinal plants, natural products can be considered a good alternative for synthetic chemical substances. Phytotherapeutic drugs have been frequently used in the treatment of a myriad of diseases, proving to be an alternative to synthetic chemicals, and have been intensively studied in the prevention of sicknesses, including oral diseases (Pai et al. 2004; Newman & Cragg 2007). Some studies have shown antifungal activity against *C. albicans* from a wide variety of extracts obtained from plants (Casaroto & Lara 2010).

Therefore, these herbal drugs may have an outstanding performance in DS treatment.

*Equisetum giganteum* L. (Equisetaceae), commonly known as cavalinha, cola de caballo, horsetail, or giant horsetail, is an endemic to Central and South America and is used in traditional medicine. For example, all over Brazil and Argentina, it is commercialized as raw material for herbal medicines and as a food supplement (Francescato et al. 2013).

Studies showed its antimicrobial activity against *Streptococcus pyogenes*, *Bacillus cereus*, *B. subtilis, Enterococcus faecalis*, *Staphylococcus aureus* and *S. epidermidis* (Kloucek et al. 2005). Thus, its antimicrobial effects may qualify the extract as a promising alternative for topical treatment and prevention of oral candidiasis (Kloucek et al. 2005; Alavarce et al. 2015). Previously, we showed that heat-cured acrylic resin specimens immersed in *E. giganteum* extract presented antiadherent activity over the initial *C. albicans* biofilm, based on quantification of biofilm mass by confocal laser scanning microscopy (Alavarce et al. 2015).

This new study evaluated the antimicrobial/antiadherent action of *E. giganteum* extract against a 24 h intermediate biofilm of *C. albicans*, considered more resistant to antimicrobial action, since the amount of extracellular polymeric substances (EPS) is greater than in initial biofilms. It has been reported that the higher the amount of EPS, the greater the microbial resistance (Gilbert et al. 1997), due to the difficult drug diffusion through this matrix. Besides, the present study investigated the viability of fungal biofilm formed *in vitro*, by counting the colony-forming units per unit area of acrylic specimens (colony-forming units (CFU)/cm^2^) in agar, and the total biomass using crystal violet assay (CV).

## Materials and methods

### Plant material and extract preparation

The *E. giganteum* aerial parts were collected in July 2012 at Jardim Botânico Municipal de Bauru, SP, Brazil (22°20′30′S and 49°00′30′W). The plant was identified at the Herbarium of UNESP, São Paulo State University "Júlio de Mesquita Filho", UNBA (Bauru, SP, Brazil), where a voucher specimen was preserved under reference number 5795. The hydroethanolic extract (70% EtOH) of *E. giganteum* was prepared as previously described by Alavarce et al. (2015). 

### Specimens preparation

For this evaluation, 240 samples of heat-cured acrylic resin (VipiCrilplus, type 1, class 1, VIPI, Pirassununga, São Paulo, Brazil) initially obtained using silicone patterns from an acrylic matrix were fabricated, with rectangular shape, measuring 10 × 10 × 4 mm. The specimens were polymerized in digital water bath at 73 °C for 90 min followed by heating for 30 min at 100 °C. To obtain a roughened surface, one surface of each acrylic resin specimen was chosen and manually abraded with high-grade sandpaper (P80; Norton Abrasivos, São Paulo, Brazil) (Verran & Maryan 1997). Surface roughness was measured using a roughness tester (Hommel Tester T 1000 basic; Hommelwerke GmbH, Schwenningen, Germany). Readings were taken at three different positions on the roughened surface of each acrylic resin specimen (Rahal et al. 2004). The surface roughness (Ra) value for all acrylic resin specimens was determined between 3 and 4 μm, able to facilitate the fungal adhesion to the acrylic resin surface (Quirynen & Bollen 1995). Thereafter the acrylic resin specimens were ultrasonicated (Ultrasonic Cleaner, Arotec, Odontobra´s, São Paulo, Brazil) for 20 min (Pereira-Cenci et al. 2007) to remove any debris from the surfaces, washed in sterile distilled water, dried and sterilized with ethylene oxide (Acecil – Comércio e Esterilização a Óxido de Etileno, Campinas, Brazil).

### Treatment protocols

The acrylic resin specimens were randomly assigned to seven groups of separated treatments: E50, E25, E16, E8, E4 (Alavarce et al. 2015), obtained by brushing 50, 25, 16, 8 and 4 mg (respectively) of 70% EtOH extract of *E. giganteum* over the surface of heat-cured acrylic resin specimens; CTRL/PBS (negative control) was obtained by immersion of specimens into sterile phosphate-buffered saline, containing 1.05 mM monobasic potassium phosphate, 155.17 mM sodium chloride and 2.966 mM dibasic sodium phosphate (PBS, pH 7.2, Gibco, Grand Island, NY); and CTRL/NaOCl (positive control) was obtained by immersion of specimens into 1% sodium hypochlorite solution (Specifica Pharmacy, Bauru, Brazil) for 10 min (da Silva et al. 2011; Alavarce et al. 2015). The treatments were performed in a 24-well tissue culture plate, which was incubated at room temperature. After treatments, each acrylic resin specimen was aseptically removed and washed three times with PBS to remove residual solutions.

### Inoculum and growth conditions

*Candida albicans* (SC5314) frozen culture stocks at 80 °C were incubated in tryptic soy broth (TSB) (Accumedia Manufactures, Inc, Lansing, MI) for 24 h, at 37 °C, under 180 rpm for reactivation. Afterwards, cells were harvested, washed with PBS, and standardized to 1 × 10^7^ cells/mL in PBS (Chandra et al. 2001).

### Biofilm development

The acrylic resin specimens with the rough side up were placed in a 24-well tissue culture plate (TPP; Trasadingen, Switzerland) containing 1 mL of standardized cell suspension. The acrylic specimens were incubated for 90 min, at 37 °C, under 75 rpm (adhesion period). After that, non-adherent organisms were removed by washing with 1 mL PBS and acrylic resin specimens were incubated in new wells for 24 h, at 37 °C, under 75 rpm to allow biofilm development, submersed in 1 mL RPMI-1640 (Sigma-Aldrich, St. Louis, MO) (Alavarce et al. 2015).

### Biofilm analysis

#### Colony-forming units

After 24 h of biofilm development, specimens were placed into a polypropylene tube containing 1 mL of sterilized PBS. Adherent microorganisms were removed from the specimens by ultrasonication (Ultrasonic Cleaner, Arotec, Odontobra´s, São Paulo, Brazil) for 20 min (Marra et al. 2012; Dantas et al. 2014). The sonicated solutions were serially diluted in PBS (10^−1^ to 10^−4^), and 50 μL of 10^−4^ dilution were plated in duplicate on Sabouraud Dextrose agar (Accumedia Manufactures, Inc, Lansing, MI). The plates were incubated at 37 °C under aerobic conditions for 24 h. After this period, the colony-forming units were counted, transformed into actual counts based on known dilution factors, and the total number of CFUs per unit area (Log_10_ CFU/cm^2^) of acrylic specimens was counted. Each group included three specimens, and the experiment was repeated four times.

#### Crystal violet

The CV staining assay was performed according to the protocol used by Peeters et al. (2008), with minor modifications. After 24 h of biofilm development, 1 mL of 99% methanol (Merck Millipore, Billerica, MA) was added for 15 min for fixation of biofilms. After that, supernatants were removed and the plates were air-dried. Then, 2 mL of a 0.1% CV solution (Sigma-Aldrich, St. Louis, MO) was added to all wells. After 20 min, the excess CV was removed by washing the plates in sterile distilled water. Finally, bound CV was released by adding 2 mL of 95% ethanol (Synth, Diadema, São Paulo) (Li et al. 2003). The absorbance was measured at 570 nm and standardized in relation to the area of acrylic specimens (Abs/cm^2^). All steps were carried out at room temperature. Each group included two specimens, and the experiment was repeated six times.

### Statistical analysis

Data obtained from CFU and CV assays were presented as means and standard deviations and statistically analyzed by Kruskal–Wallis test or ANOVA, complemented by Miller test or Tukey test, respectively, at a significance level of 5%, on the statistical software Prisma 6.0 (San Diego, CA). 

## Results

### Colony-forming units

**Figure 1.**
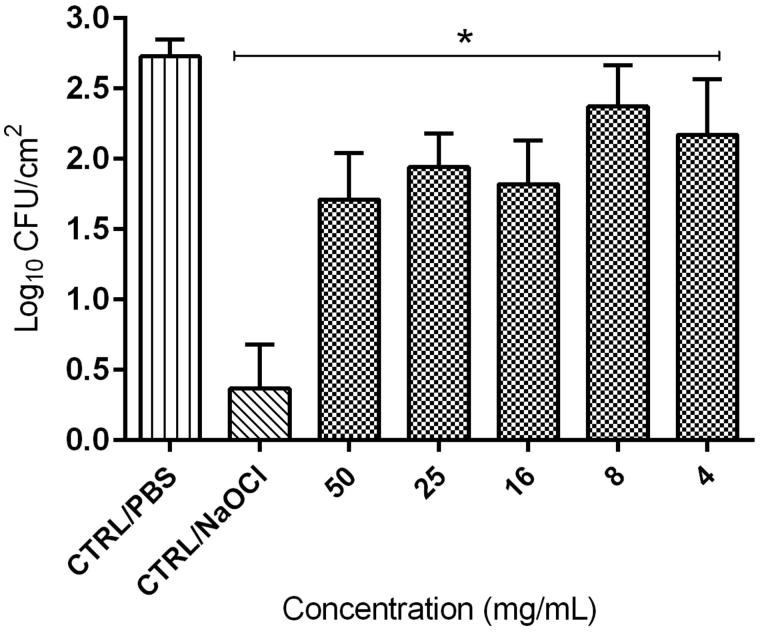
Antifungal activity of *E. giganteum* extracts against *C. albicans*. Mean ± S.D. of viable cells (Logarithm of colony forming units per cm^2^) remaining on the surface of resin specimens after different pretreatments with extract: 4–50 mg/mL, or PBS (CTRL/PBS) or 1% sodium hypochlorite (CTRL/NaOCl), for 10 min. Data were obtained from analysis of 12 specimens for each group. *p < 0.05 compared to the control (CTRL/PBS).

The resin pretreatment with *E. giganteum* extract (50, 25, 16, 8 and 4 mg/mL) showed fewer colonies compared to the negative control (CTRL/PBS) (Log_10_ CFU/cm^2^). These results showed cell density reduction of up to 79% in relation to CTRL/PBS (Table 1). As shown in Figure 1, 70% EtOH extract of *E. giganteum* in all concentrations demonstrated significant antibiofilm activity (*p* < 0.05) against *C. albicans*, and this result was statistically similar to 1% NaOCl. No growth inhibition was observed in the negative control (CRTL/PBS). Only the 8 mg/mL concentration was not statistically similar to CTRL/NaOCl, presenting a slightly higher number of colonies in comparison to the other extract concentrations. Yet, it presented a 50% reduction in relation to CTRL/PBS.

**Table 1.** Comparison of results from CV and CFU assays, and reduction of viable cells in relation to Control (CTRL/PBS), observed in biofilms remaining on the surface of the resin specimens after different pretreatments with extract: 4–50 mg/mL, or PBS (CTRL/PBS) or 1% sodium hypochlorite (CTRL/NaOCl), for 10 min.

		
Treatment (Groups)	Biofilm mass determinationCV Abs(570nm)/cm^2^ ± SD	Log_10_ CFU/cm^2^ Reduction(% in relation to CTRL/PBS)
E50	1.55 ± 1.11	1.71 (79)
E25	1.41 ± 0.39	1.94 (74)
E16	1.67 ± 0.42	1.82 (74.5)
E8	2.00 ± 0.70	2.38 (50)
E4	2.12 ± 0.86	2.17 (66)
CTRL/NaOCl	0.79 ± 0.58	0.37 (99.5)
CTRL/PBS	2.51 ± 0.60	2.73 (0)

### Crystal violet

**Figure 2.**
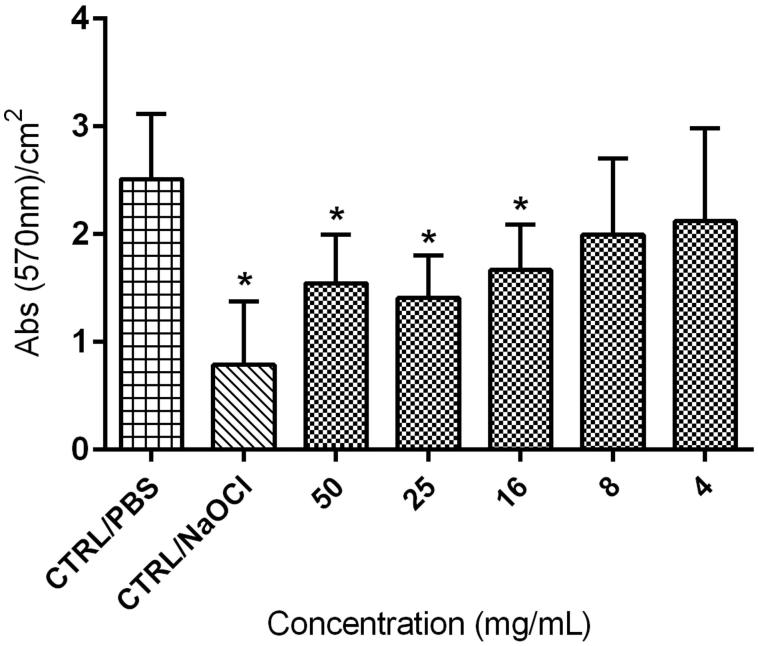
Mean absorbance values per cm^2^ obtained by CV assay on biofilms remaining on the surface of resin specimens after different pretreatments with extract: 4–50 mg/mL, or PBS (CTRL/PBS) or 1% sodium hypochlorite (CTRL/NaOCl), for 10 min. *p < 0.05 compared to the control (CTRL/PBS).

Total biofilm mass assessment using CV staining demonstrated that *E. giganteum* allowed formation of *C. albicans* biofilm regardless of extract concentration. However, differences occurred depending on the concentration (Table 1). As shown in Figure 2, statistical significance was observed at the highest concentrations of extract (50, 25 and 16 mg/mL) and 1% NaOCl, compared to CTRL/PBS. These results have shown reduction of 15–44% (from 2.12 to 1.41 absorbance) of biofilm compared to the untreated control (CTRL/PBS).

## Discussion

The growth of biofilms on the denture base is a critical aspect in the development of denture stomatitis, a type of oral candidiasis that affects denture wearers, especially individuals wearing maxillary complete dentures (Zomorodian et al. 2011). Over-the-counter topical antifungal drugs are often unable to eliminate microorganisms present on the denture base, while the systemic use of antifungals could result in side effects, e.g. hepatotoxicity, nephrotoxicity, and increased microbial resistance (Lombardi & Budtz-Jorgensen 1993).

Although there are few approaches about the influence of herbal medicines on *C. albicans* viability, especially on acrylic resins (Casaroto & Lara 2010), and a scarcity of published studies about *E. giganteum* biological activities, it is important to mention that it is an easy-to-find and abundant native species of some countries, such as Brazil (Alavarce et al. 2015; Kloucek et al. 2005). A research that used an *in vitro* model similar to our study concluded that resin surface pretreatment with traditionally used antimicrobial agents significantly reduced the adhesion of *Candida* species on acrylic resin (Mccourtie et al. 1986), thereby demonstrating that pretreatment of acrylic resin with antimicrobial substances can be effective.

In a previous *in vitro* study, we demonstrated that this herbal drug has antimicrobial activities against *C. albicans*, *S. aureus*, and *E. coli*; antiadherent action over *C. albicans* into heat-cured acrylic resin specimens model; and anti-inflammatory effects on human monocytes activated by *C. albicans* without compromising the cell viability (Alavarce et al. 2015). In this initial study, the antiadherent and antimicrobial assays were performed against a 12-h initial biofilm of *C. albicans,* and the anti-inflammatory and cytotoxicity tests used experimental models based on extract immersion on human cells (Alavarce et al. 2015), where the extract did not show cytotoxicity in contact with these cells.

In the current study, the antiadherent activity of *E. giganteum* extract against *C. albicans* was assessed in a 24 h biofilm, in which the amount of EPS is greater, which has been reported as one of the factors influencing the occurrence of microbial resistance. Some studies suggest that EPS present in microbial biofilms physically interact with antimicrobial drugs contributing to microbial resistance. However, for other authors, it is unclear if this resistance to antimicrobial drugs is due to production of EPS or due to genetic and biochemical changes in fungal cells (Hoyle & Costerton 1991; Gilbert et al. 1997). Besides, in the present work, the extract was brushed over the resin specimens and not immersed, which resulted in higher amount of residual extract on the surface than in immersed specimens.

CFU and CV assays were the two methods selected for this study. The main difference between CV and CFU is that the former stains both live and dead cells, together with the EPS, allowing quantification of total biofilm biomass (Hawser 1996; Ramage et al. 2001). Besides, it is a cheaper and faster method. Since the CV assay stains negatively charged molecules (live and dead cells) on the surface, as well as the polysaccharides that compose the extracellular matrix, it cannot be considered a suitable assay to assess the antibiofilm efficacy of herbal or any other antimicrobial substances (Pantanella et al. 2013).

CFU, a gold standard in microbiology, analyzes the viability of biofilm mass and must be carried out after biofilm disruption. It is a hardworking and time-consuming method (Donlan & Costerton 2002). The CFU was used to complement data obtained from the CV assay, providing quantification of biofilm living cells. Since they are complex structures, studies on biofilms require multiple approaches that characterize their different aspects, which cannot be obtained by a single assay (Peeters et al. 2008).

Our results showed that the resin surface previously treated by brushing with *E. giganteum* extract at all concentrations produced significant reduction of *C. albicans* biofilm mass compared with the untreated resin (CTRL/PBS). This was most evident from the concentration of 16 mg/mL. In the highest concentration, the reduction in biofilm cell viability was 79%.

Results obtained from both methods of biofilm analysis were compared and correlated, because in general the lower number of viable cells counted in the biofilm (CFU assay) matched a minor amount of extracellular matrices remaining on the acrylic resin surface (CV assay). Therefore, we suggest that, for this reason, the highest CFU values (extract concentrations 8 and 4 mg/mL) in CV assay showed no statistical difference with the negative control (PBS). From these two methods, it was possible to verify that the extract brushed on the surface of specimens was able to reduce not only the number of remaining viable cells, but also the amount of EPS.

Because of the multifactorial process of biofilm formation and the complexity of bioactive substances that form the crude extract, this investigation cannot state the exact mechanism and/or factor responsible for the antiadherent effect of *E. giganteum* on *C. albicans* biofilms, since this extract is able to act on different targets using different mechanisms for biofilm development (Braga et al. 2008). However, we previously verified that *E. giganteum* extract presents in its composition phenolic compounds derived from caffeic and ferulic acids and flavonoid heterosides derived from quercetin and kaempferol, which are known to have antimicrobial activity, because of the ability to inactivate adhesion proteins and cause disruption of the microbial cell membrane (Alavarce et al. 2015; Cowan 1999). Another possibility that should be taken into account, regarding the antiadherent effect of the extract on *C. albicans*, would be that the extract can interact and modify the surface of heat-cured acrylic resin, increasing the contact angle and reducing its surface free energy, making it more hydrophobic, which decreases the fungal adhesion (Al-Dwairi et al. 2012; Combe et al. 2004).

This study was done with the extract in its raw form, which is a complex mixture represented by both primary and secondary metabolites. In general, isolation of the active principles of interest (fractions usage) results in better outcomes from lower concentrations of the herbal extracts (Machado et al. 2003).

Thus, we suggest that, by isolation of active compounds of the raw extract (fractions usage) we can obtain a more effective antifungal/antimicrobial activity in lower concentrations than with the extract (Wolfender et al. 2015).

## Conclusions

These results suggest that *E. giganteum* antimicrobial potential and its ability to eliminate or prevent the adhesion of *C. albicans* to resin surface may qualify the extract as a promising natural alternative for topical treatment or prevention of denture stomatitis. After further testing, it will be able to be clinically applied in microorganisms-related infectious diseases, such as DS.
